# Guilt, Disgust, and Not Just Right Experience Mediate the Effect of Demanding Parent Mode on Obsessive-Compulsive-Disorder-like Tendencies, and Punitive Parent Mode Moderates This Mediation

**DOI:** 10.3390/bs13090700

**Published:** 2023-08-23

**Authors:** Suzana Semeniuc, Ancuța Elena Păduraru, Camelia Soponaru

**Affiliations:** Department of Psychology, “Alexandru Ioan Cuza” University of Iași, 700506 Iași, Romania; suzana.semeniuc@yahoo.com (S.S.); anca.paduraru@uaic.ro (A.E.P.)

**Keywords:** obsessive compulsive disorder, guilt, demanding parent mode, punitive parent mode, schema modes

## Abstract

The main objective of the present study was to examine, in a non-clinical population, the validity of a moderated mediation model for obsessive mental functioning. The research was conducted on a sample of 205 participants. Data were collected using the Psychiatric Screening and Diagnostic Questionnaire, Young’s Modes Questionnaire-form SMI-2, Padua Inventory of Obsessive-Compulsive Symptoms, Fear of Guilt Scale in OCD, Disgust Propensity and Sensitivity Scale-Revised, and Not Just Right Experiences Questionnaire-Revised. The results revealed that there is a significant positive, indirect effect of the Demanding Parent mode on OCD-like tendencies, which is completely mediated by fear of guilt, tendency and sensitivity to disgust, and Not Just Right Experiences severity. The Punitive Parent mode moderates only the indirect effect of the Demanding Parent mode mediated by fear of guilt, not the indirect effect mediated by disgust tendency and sensitivity and Not Just Right Experiences severity. Also, the indirect effect of the Demanding Parent mode on obsessive tendencies mediated by fear of guilt is significant only at medium and high values of the Punitive Parent mode, not at low values of the moderator. Our results provide a novel direction targeting the direct therapeutic intervention on demanding and punitive internal dialogue, complementing the classical CBT intervention protocol.

## 1. Introduction

Intrusive, ego-dystonic mental thoughts or images are common experiences for most of us [1]. When, however, more and more mental energy and time are consumed in removing them from the awareness level through various mental or behavioral compulsions, the person reaches the clinical cutoff for obsessive compulsive symptoms, a disorder that affects a significant proportion of the population [2,3].

Obsessive compulsive disorder (OCD), characterized by the presence of obsessive thoughts/images perceived as egodystonic and compulsions designed to reduce the immediate discomfort they produce (American Psychiatric Association, 2013), affects a significant proportion of the population [2,3]. Although both obsessions and compulsions are qualitatively common for most people [1], a proportion of us end up suffering from obsessive compulsive spectrum disorders, the frequency of occurrence being the key factor that makes the difference in the transition to pathology. There is a consistent body of empirical evidence for the presence of excessive blame and guilt among the clinical population as opposed to the non-clinical [4,5,6,7,8]. Also, a number of experimental studies have concluded that the escalation of the two has the effect of increasing OCD-like behaviors in the non-clinical population [9,10,11,12,13], and other neuroimaging studies [14,15] have revealed an overlap between brain areas involved in guilt processing and those affected by OCD. 

Mancini [1] proposed a refinement of the concept of guilt distinguishing between deontological and altruistic guilt. The former implies the violation of a rule in the person’s value system, and the latter the necessary existence of a victim [1,16,17]. The cognitive model of obsessive disorders proposed by Mancini [1] finds as its central assumption that the primary goal of OCD sufferers is to prevent deontological guilt, and a potential failure to achieve this goal “is perceived as an unacceptable and unbearable catastrophe” [1] (p. 51). Apart from this, the model includes two other important goals of OCD sufferers, namely the neutralization of disgust and the feeling of “not just right experience” (NJRE)—the result of a mismatch between perception and reference schema, which can be felt at the level of all analyzers [18]. Thus, an event (trigger) is interpreted by the subject as a threat, depending on the first evaluation (contamination/NJRE or the possibility of being guilty in the deontological sense). The interpretation leads to Attempted Solution 1—a complex, automatic reaction composed of emotions (anxiety, disgust, fear of guilt), specific cognitive processes (selective attention and memory), and observable or mental behaviors (compulsions). This reaction, over time, has a paradoxical effect—it leads to generalization (trigger situations occur more often, confirm the belief about the threat, lead to a greater investment of time and energy in attempts to resolve it, and thus increase the value of the threatened goal). Meta-evaluation comes next, or second-order evaluation, usually self-derogatory and critical of the first-order evaluation and solution attempt [1]. 

The Schema Therapy (ST) model defines early maladaptive schemas as dysfunctional ways of organizing information about the self and others, consisting of memories, emotions, cognitions, and bodily sensations, developed as a result of repeated experiences of frustration with basic emotional needs in childhood and adolescence [19]. The concept of the schema mode refers to a specific mental state that arises from the activation of one or more schemas. There are several types of modes that describe the intrapsychic dynamics of the person: child modes (parts that express unaddressed needs: Vulnerable child—lonely, guilty, abused, neglected, sad—Angry child, Impulsive child), dysfunctional parent modes (parts that represent introjected parental messages—Punitive Parent, Demanding Parent), and specific coping modes (Surrender modes—compliant, less assertive—Detached/Avoidant modes, Overcompensation modes) [20,21]. In terms of early maladaptive schemas, there does not seem to be a specificity of OCD sufferers, as they report higher scores compared to other pathologies across a range of maladaptive schemas including Dependence/Incompetence, Vulnerability to hazards, Abandonment, Social Isolation, Emotional Deprivation, Entitlement, Subjugate, Search for validation, Negativity/Pessimism, and Unrealistic Standards [22,23,24,25,26]. In terms of schema modes, however, a number of studies have identified a specific organization of obsessive functioning that includes Vulnerable child mode (fearful and guilty), Angry child mode, Punitive Parent mode, Demanding Parent mode, Perfectionist Over-controller mode, and Detached Self-soother mode [25,26,27,28,29]. Some research [20] has attempted to integrate the two cognitive models into a common conceptualization with the aim of developing more effective therapeutic interventions for OCD sufferers. Building on these theoretical attempts, we set out to empirically test a moderated mediation model based on these two representations.

**Figure 1:**
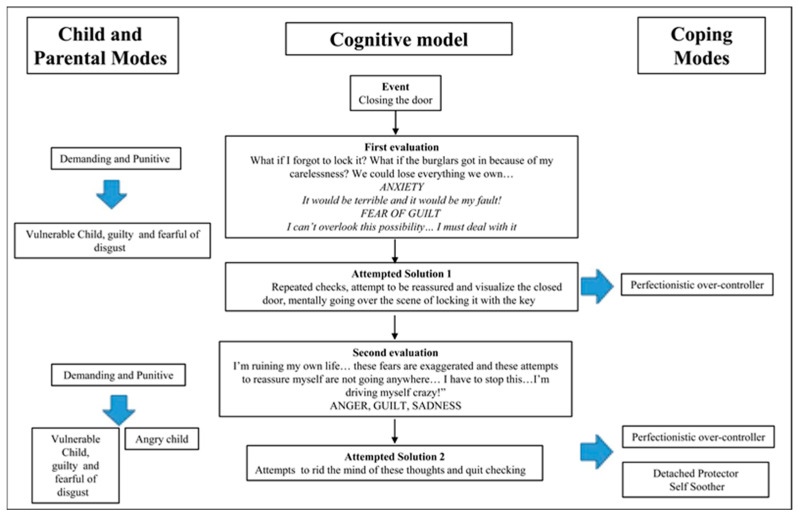
An integration of schema and cognitive therapy in OCD treatment [20].

Consistent with the review of the two cognitive models as well as the common conceptualization of OCD proposed by Tenore [20], the main objective of the present study is to examine, in a non-clinical population, the validity of an integrated, moderated mediation explanatory model for obsessive-like psychological functioning. Our hypothesis builds on previous attempts in the literature [20] (pp. 2278–2295) to bring together the two cognitive models, namely the Mancini model and the ST model, into a common conceptualization, summarized in the Figure 1 exemplified below:

The new aspect of our article is that it differentiates between the role of the demanding parent (who imposes unrealistic rules and standards) and the critical and punitive parent (who punishes) in maintaining obsessive mental dynamics. 

Thus, we propose as the first Hypothesis (1) that fear of guilt, disgust tendency and sensitivity, as well as NJRE severity mediate the effect of the Demanding Parent mode on the level of OCD-like tendencies, and the second Hypothesis (2) is that this mediation is moderated by the Punitive Parent mode.

## 2. Materials and Methods

### 2.1. Participants

This study involved 348 adult subjects, aged between 19 and 59. Inclusion criteria were respondents over 18 years of age, Romanian, and not currently undergoing medication or psychotherapeutic treatment for a psychiatric diagnosis. The exclusion criteria from the research group were clinical-level symptoms at screening for depression, psychotic disorders, panic disorder, and generalized anxiety disorder. Respondents were recruited among students at universities in Iasi, as well as through advertisements and social networks. Of these, 143 participants were screened out because they met clinical scores for a range of mental disorders (depression—91, psychotic disorders—18, panic disorder—13, generalized anxiety—21). Thus, the research was carried out on a group of 205 participants including 173 women and 32 men, 148 from urban and 57 from rural backgrounds, and 38 with a psychiatric history in the primary family and 167 without such a history (Table 1). All participants were assured confidentiality of their responses (data were only accessed by the research team) and were treated in accordance with the standard ethical rules of the faculty to which the researchers belong and of the Declaration of Helsinki. This study was approved by the Ethics Committee of the Faculty of Psychology and Educational Sciences of “Alexandru Ioan Cuza” University of Iași.

### 2.2. Instruments

All questionnaires were completed via an online survey and were anonymous.

First, the subjects completed a demographic questionnaire. Through this questionnaire, we obtained data on the biological gender of the subjects, age, background, level of education (high school, university, postgraduate), and existence of psychiatric history of any kind in the primary family.

The Psychiatric Screening and Diagnostic Questionnaire (PDSQ) [30] Scale with 111 items (each with values of 0 or 1) measures clinical-level symptoms for 13 Axis I psychiatric disorders and has been adapted for the Romanian population. We used only the subscales necessary to verify the identified confounding variables that we controlled by design (the most common psychiatric comorbidities for OCD), namely depressive disorder, psychosis, panic disorder, and generalized anxiety [31]. Thus, the subscales for depression (21 items, section score 9), panic disorder (8 items, section score 4), psychotic disorders (6 items, section score 1), and generalized anxiety (10 items, section score 8) were applied.

Young’s Modes Questionnaire-form SMI-2 [32]: This instrument has 143 items organized into 16 subscales, which measure 16 modes (each mode is described by items that address specific emotions, cognitions, and behaviors) asking subjects to rate frequency on a six-category ordinal scale (from “0-never or almost never” to “6-always”). A high score on a subscale measuring a mode represents a frequent manifestation of that mode in the individual’s life. In our research, we used subscales that measure modes that occur in the conceptualization of obsessive compulsive disorder and are the subject of our hypotheses. Thus, we used subscales measuring the dysfunctional parent mode, namely the subscales for Demanding Parent and Punitive Parent. Original psychometric subscale data were not available for this inventory.

Padua Inventory of Obsessive-Compulsive Symptoms [33]: This instrument contains 39 items rated on a five-step Likert scale (1—Not at all, 5—Very much) and has been used in numerous studies of obsessive compulsive symptoms in non-clinical populations. The internal consistency coefficient α-Cronbach of the scale, reported by authors, was 0.92.

Fear of Guilt Scale for OCD (Fear of Guilt Scale for OCD) [34]: This instrument contains 17 items describing two factors (Punishment—the tendency to punish oneself for feeling guilty, and Avoidance—the tendency to avoid experiencing guilt) and is rated by the subject on a seven-category ordinal scale (from total disagreement—1 to total agreement—7). The scale was constructed to measure fear of guilt specific to OCD. The authors note that the benefit their instrument brings is the possibility of empirically testing the Mancini model because the scale aims to measure specific guilt fear, corresponding to the deontological guilt in the Mancini model, which causes individuals to be concerned about their conduct in a situation rather than the consequence of the action itself [34]. In the original study, the internal consistency coefficient α-Cronbach of the scale was 0.92

Disgust Propensity and Sensitivity Scale-Revised [35]: This instrument contains 16 items measuring two factors, disgust sensitivity and disgust proneness. The items are rated on a 5-step Likert scale (1—never, 5—always), and the score is calculated separately on the two subscales. The instrument has also been validated for clinical populations on the obsessive disorder spectrum. The internal consistency coefficients α-Cronbach calculated by the authors on each subscale were 0.73 (disgust proneness) and 0.60 (disgust sensitivity), and for the total scale, the internal consistency coefficient was 0.70 [36].

Not Just Right Experiences Questionnaire-Revised (NJREQ-R) [37]: This instrument contains 17 items consisting of 10 statements, each of which requires subjects to rate on a seven-step Likert scale the frequency, intensity, need to intervene, immediate and subsequent distress, rumination, and responsibility. The total score on the last seven items indicates the severity of the NJRE. The internal consistency coefficient α-Cronbach of the scale for the original group was 0.67.

### 2.3. Statistical Analysis

All analyses were conducted using IBM SPSS 28 for Windows (IBM Corporation, Armonk, NY, USA). To test the two hypotheses, we used the methodology proposed by Hayes [38], using PROCESS (model 4 and model 14, 2013). In line with the common conceptualization of the two cognitive models, the independent variable was the Demanding Parenting mode (internal critical dialogue marked by unrealistic rules and standards); the mediators were fear of guilt, disgust propensity and sensitivity, and not just right experience (in line with the Mancini model, which supports the three dimensions as the main determinants of obsessive mental functioning); and the dependent variable was the level of obsessive compulsive symptomatology. Although the common conceptualization does not differentiate the roles of the Demanding and Punitive Parent modes in obsessive-like psychic functioning, we propose the Punitive parent mode (critical and punitive internal dialogue) as a moderator.

## 3. Results

We tested the internal consistency of the scales used in our study by calculating Cronbach’s α-coefficient. For Young’s Modes Questionnaire-form SMI-2, the internal consistency coefficients α-Cronbach were calculated for each subscale and were 0.78 (Punitive Parent) and 0.80 (Demanding Parent). The Padua Inventory of Obsessive-Compulsive Symptoms for our sample had an internal consistency coefficient α-Cronbach equal to 0.94. For the Fear of Guilt Scale for OCD, the internal consistency coefficient Cronbach’s α was 0.87. The Cronbach’s α internal consistency coefficients were calculated on each subscale for the Disgust Propensity and Sensitivity Scale-Revised and were 0.86 (disgust proneness) and 0.86 (disgust sensitivity), and for the total scale, the coefficient was 0.91. For the Not Just Right Experiences Questionnaire-Revised, we obtained an internal consistency coefficient α-Cronbach equal to 0.92.

The normality assumption did not meet the distribution of all variables, which is why we used a bootstrap approach for data analysis [39]. All the information can be found in Table 2.

**Figure 2:**
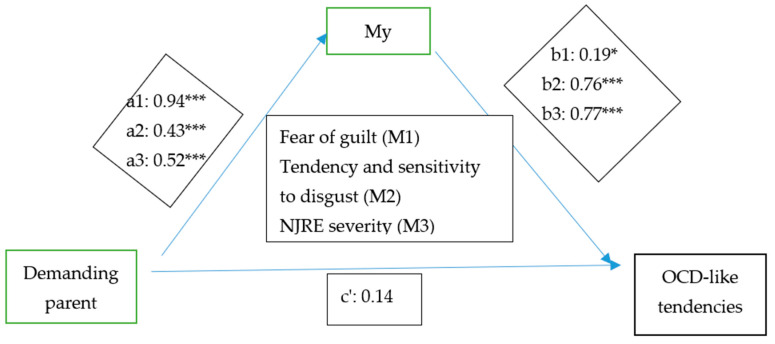
Effect of Demanding Parent mode on OCD tendencies mediated by fear of guilt, disgust tendency and sensitivity, and NJRE. *: *p* = 0.01; ***: *p* < 0.001.

To test the first Hypothesis (1) of our study, that fear of guilt, tendency and sensitivity to disgust, together with NJRE severity mediate the effect of the Demanding Parent mode on the level of OCD tendencies, we used PROCESS (model 4) proposed by Hayes [38] (Figure 2). 

The results show that the Demanding Parent mode predicts fear of guilt (b = 0.94, t(203) = 8.13, *p* < 0.001). Also, the Demanding Parent mode is a significant predictor of disgust tendency and sensitivity (b = 0.43, t(203) = 4.63, *p* < 0.001) and NJRE severity (b = 0.52, t(203) = 6.47, *p* < 0.001). 

In turn, the three mediators are significant predictors of OCD-like tendencies. Thus, fear of guilt predicts obsessive tendencies (b = 0.19, t(200) = 2.40, *p* = 0.01); tendency and sensitivity to disgust is also a significant predictor of OCD-like tendencies (b = 0.76, t(200) = 7.44, *p* < 0.001), as is NJRE severity (b = 0.77, t(200) = 6.41, *p* < 0.001).

The overall effect of the Demanding Parent mode on OCD-like tendencies is positive and significant (b = 1.06, t(203) = 6.13, *p* < 0.001) (Table 2). There is no direct, significant effect of the Demanding Parent mode on obsessive tendencies (b = 0.14, t(200) = 1.00, *p* = 0.31), implying that the mediation is total. The results, summarized in Table 3, indicate that our hypothesis is confirmed, such that fear of guilt mediates the effect of the Demanding Parent mode on OCD-like tendencies (indirect effect = 0.18, SE = 0.09, 95% CI (0.0061; 0.3712)—CI does not include 0), along with disgust tendency and sensitivity (indirect effect = 0.33, SE = 0.08, 95% CI (0.1830; 0.5178)—CI does not include 0) and NJRE severity (indirect effect = 0.40, SE = 0.09, 95% CI (0.2315; 0.5958)—CI does not include 0).

We can therefore conclude from the results that there is a statistically significant indirect effect of the Demanding Parent mode on OCD-like tendencies, and that it is mediated entirely by fear of guilt, tendency and sensitivity to disgust, and NJRE severity.

**Figure 3:**
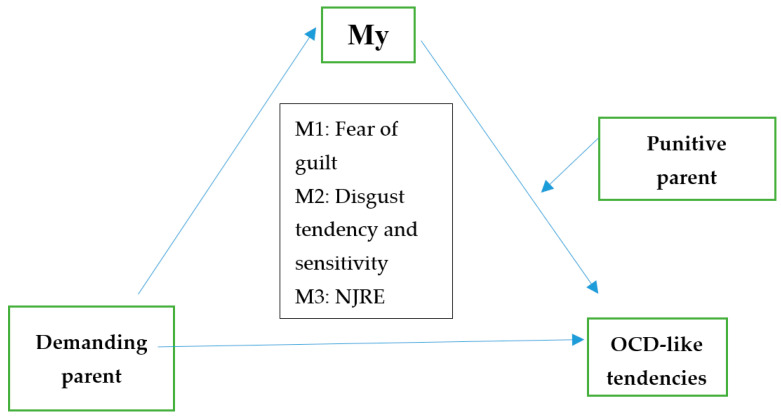
A moderated mediation explanatory model for obsessive mental functioning.

To test Hypothesis (2) of the present study, that the mediation of the effect of the Demanding Parent mode on OCD tendencies by the fear of guilt tendency and sensitivity to disgust and NJRE severity is moderated by the Punitive Parent mode, we used PROCESS (model 14) proposed by Hayes [38] (Figure 3). 

The results show that the Punitive Parent mode significantly moderates only the effect of fear of guilt on OCD-like tendencies (βsimple = 0.04, SE = 0.01, *p* = 0.01, 95% CI (0.0086; 0.0724)—CI does not include 0), not the effect of disgust tendency and sensitivity or NJRE severity on obsessive tendencies (Table 4). 

**Figure 4:**
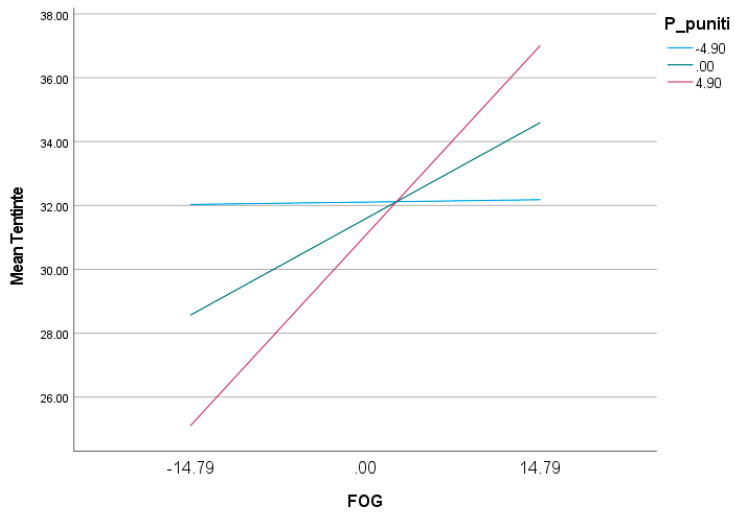
Effect of fear of guilt on obsessive tendencies to different values of Punitive Parent mode.

At low Punitive Parent mode scores (one standard deviation below the mean), the effect of fear of guilt on OCD-like tendencies is positive but statistically insignificant (b = 0.005, SE = 0.10, *p* = 0.96, 95% CI (−0.2023; 0.2124)—CI includes 0). In contrast, as Punitive Parent mode scores increase, the effect of fear of guilt on obsessive tendencies also increases and becomes significant. Thus, at high Punitive Parent mode scores (one standard deviation above the mean), the effect of fear of guilt on OCD-like tendencies is positive and statistically significant (b = 0.40, SE = 0.12, *p* = 0.001, 95% CI (0.1575; 0.6473)—CI does not include 0) (Table 5; Figure 4).

**Figure 5:**
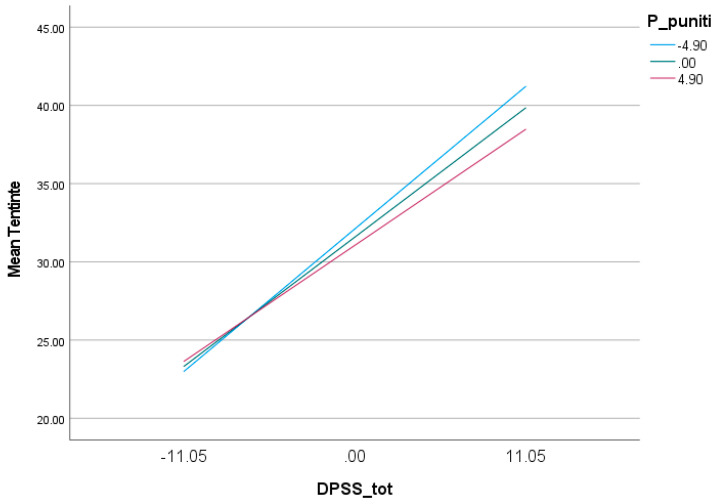
Effect of disgust tendency and disgust sensitivity on obsessive tendencies at different values of Punitive Parent mode.

The Punitive Parent mode does not significantly moderate the effect of disgust tendency and sensitivity on obsessive tendencies (Table 4, Figure 5).

**Figure 6:**
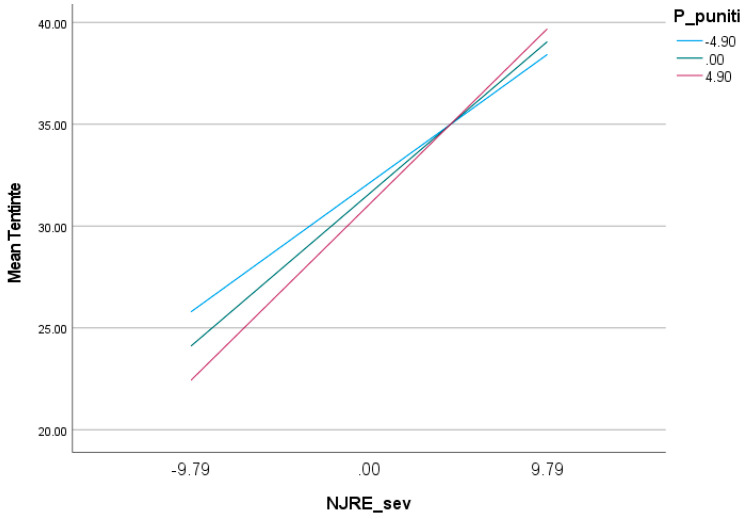
Effect of NJRE severity on obsessive tendencies at different values of Punitive Parent mode.

Also, the Punitive Parent mode does not significantly moderate the effect of NJRE severity on OCD-like tendencies (Table 4, Figure 6).

The bootstrap results also show a significant moderated mediation effect in which the Punitive Parent mode moderates the indirect effect of the Demanding Parent mode on obsessive tendencies, mediated by fear of guilt. Thus, at low values of the Punitive Parent mode (one standard deviation below the mean), the indirect effect of the Demanding Parent mode on OCD-like tendencies mediated by fear of guilt is positive but statistically insignificant (ind effect = −0.004, SE = 0.10, 95% CI (−0.2077; 0.2156)—CI includes 0). At high moderator values (one standard deviation above the mean), the indirect effect of the Demanding Parent mode on obsessive tendencies, mediated by fear of guilt, increases and becomes statistically significant (ind effect = 0.37, SE = 0.12, 95% CI (0.1162; 0.6169)—CI does not include 0) (Table 6).

The Punitive Parent mode does not moderate the indirect effect of the Demanding Parent mode on OCD-like tendencies mediated by disgust tendency and sensitivity. The effect tends to decrease with increasing Punitive Parent mode, but not significantly (Table 7).

There is also no significant moderation on the indirect effect of the Demanding Parent mode on obsessive tendencies mediated by NJRE severity. In this case, we observe a trend of increasing indirect effect as Punitive Parent mode values increase, but this is not statistically significant (Table 8).

We can therefore conclude that the second hypothesis of our study is only partially confirmed, in the sense that the Punitive Parent mode moderates only the indirect effect of the Demanding Parent mode mediated by fear of guilt, not the indirect effect mediated by the tendency and sensitivity to disgust and the severity of NJRE. Moreover, the results of the analysis show that the indirect effect of the Demanding Parent mode on obsessive tendencies mediated by fear of guilt is significant only at medium and high values of the Punitive Parent mode, not at low values of the moderator.

## 4. Discussion

The objective of the present study was to propose and examine, in a non-clinical population, the validity of an integrated, moderated mediation model for obsessive mental functioning based on cognitive theories [1,25] and previous research that proposed a common conceptualization model [20].

The two hypotheses, formulated in line with this objective, were as follows: (1) fear of guilt, tendency and sensitivity to disgust, as well as NJRE severity mediate the effect of the Demanding Parent mode on the level of OCD-like tendencies; and (2) this mediation is moderated by the Punitive Parent mode.

The results revealed that there is a significant positive, indirect effect of the Demanding Parent mode on OCD-like tendencies and this is fully mediated by fear of guilt, tendency and sensitivity to disgust, and NJRE severity, thus supporting our first hypothesis. Fear of guilt corresponds to the concept of deontological guilt described by Mancini, in which the individual is excessively concerned with the particular mode of conduct and not necessarily with the negative outcome itself [1,34]. We can therefore conclude that, in line with previous research, a demanding and critical home environment [40,41], which induces guilt [42] or is characterized by parental control and criticism, high expectations, pressure toward unrealistic standards [43,44], and leads to the introjection of a demanding parental figure, i.e., the Demanding Parent mode [32], has an indirect effect on obsessive tendencies. The more demanding the internal dialogue, the greater the fear of guilt, the tendency and sensitivity to disgust, and the severity of the NJRE. And the higher these are, as supported by the model proposed by Mancini [1], the higher the increase in intensity of OCD-like symptomatology.

As for the second hypothesis of our study, it was only partially confirmed. Consequently, in view of previous research proposing the integration of the Schema Therapy Modes model with the Mancini model [20,45], as well as the data obtained in the first part of this study, we proposed and analyzed an integrated explanatory model of moderated mediation. Thus, we wanted to test whether the indirect effect of the Demanding Parent mode on OCD-like tendencies, mediated by fear of guilt, tendency and sensitivity to disgust, together with NJRE severity, is moderated by the Punitive Parent mode. The results suggest that there is moderate mediation only in the case of the guilt–fear-mediated relationship. Furthermore, from the data, we can state that the indirect effect of the Demanding Parent mode on obsessive tendencies is mediated by fear of guilt only in the case of medium and high values (one standard deviation from the mean) of the Punitive Parent mode; in the case of low values of this mode (one standard deviation below the mean), the indirect effect mediated by fear of guilt remains positive but is insignificant. In the case of the indirect effect of the Demanding Parent mode on OCD-like tendencies mediated by disgust tendency and sensitivity as well as NJRE severity, there is no moderated mediation by the Punitive Parent mode. The results are in agreement with previous research that argues that a Punitive Parent mode that results in a very low tolerance for fault, i.e., withdrawal of affect, ignoring the child, and thus threatening the loss of the meaningful relationship [46,47], causes guilt-experiencing, which should be avoided at all costs. In other words, if the demanding internal dialogue, with unrealistic standards and over-responsibility, characteristic of the Demanding Parent mode, (e.g., “I must do everything perfectly”/“I cannot afford to be wrong”) is coupled with a punitive, unforgiving internal dialogue, characteristic of the Punitive Parent mode (“I am a bad person”/“I do not deserve compassion”/“I deserve to be punished”, etc.), the level of obsessive-like symptoms grows significantly by increasing the level of fear of guilt.

The results indicate that the internalization of a demanding internal dialogue, centered on excessive rules and unrealistic standards, resulting from a strict parental style, excessively centered on morality, only partially explains the development of an obsessive mental functioning. What moderates the effect of such an environment and can favor the development of an OCD pathology is the consequence of deviating from rules and standards. If this consequence is applied to the child in the form of harsh criticism and punishment, internalized in the form of an internal self-critical and self-punishing dialogue, the risk of developing an obsessive compulsive pathology increases significantly. The tested model is different from other mediation models because it starts from the common conceptualization of the two cognitive models. Other models do not take into account the role of internal dialogue in the severity and maintenance of this pathology.

The results have some clinical implications, as they support previous results of experimental research [45,48,49] that a direct therapeutic intervention on demanding and punitive internal dialogue and, thus, on the Demanding Parent mode and Punitive Parent mode can lead to a decrease in OCD symptoms as well as to the long-term maintenance of the results achieved by the classical CBT intervention protocol, which exclusively targets fear of guilt, disgust proneness and sensitivity, or NJRE severity (hence mediators), in obsessive patients.

As limitations of our study, we mention mainly the impossibility of generalizing conclusions, given that our study group is not a representative sample of the non-clinical population. Also, the fact that the research group was predominantly female may have influenced the results. This limitation can be remedied by replicating this study on a more homogeneous group. It is also possible that other confounding variables that have not been controlled may influence our conclusions, such as potential personality disorders of subjects frequently associated with obsessive compulsive disorder-avoidant, dependent, borderline, obsessive compulsive, passive-aggressive, histrionic personality disorder [31]. 

We therefore believe that future research would be needed to verify our proposed mediation and moderated mediation models. Also, further research might consider testing the validity of the model on a more balanced sample and taking into account other confounding variables such as possible strong personality tendencies.

## 5. Conclusions

In conclusion, the results of our study showed that there is a significant positive, indirect effect of the demanding parenting mode on OCD-like tendencies and it is completely mediated by fear of guilt, tendency and sensitivity to disgust, and severity of NJRE. Furthermore, if demanding internal dialogue is coupled with punitive internal dialogue, it significantly increases the level of obsessive-like symptomatology by increasing the level of fear of guilt. Thus, therapeutic interventions aimed at decreasing OCD-type symptomatology, in a non-clinical population, might consider directly addressing demanding and punitive internal dialogue, specifically the Demanding Parenting mode and Punitive Parenting mode.

## Figures and Tables

**Table 1:** Sociodemographic characteristics of the sample.

Variable	Frequency	Percentage (%)
Biological gene		
Male	32	15.6
Female	173	84.4
Age		
19–35 years	156	76.1
36–50 years	40	19.5
over 50 years	9	4.4
Background		
Rural	57	27.8
Urban	148	72.2
Level of education		
High school	112	54.6
University	66	32.2
Postgraduate	27	13.2
Psychiatric history in the family of origin		
No	167	81.5
Yes	38	18.5

**Table 2:** Tests of normality.

	Kolmogorov–Smirnov ^a^			Shapiro–Wilk		
	Statistic	df	Sig.	Statistic	df	Sig.
P_punitiv	0.109	205	<0.001	0.923	205	<0.001
P_exigent	0.039	205	0.200 *	0.996	205	0.889
OCD-like tendencies	0.100	205	<0.001	0.943	205	<0.001
FOG	0.065	205	0.037	0.988	205	0.071
NJRE_sev	0.093	205	<0.001	0.960	205	<0.001
S_disgust	0.135	205	<0.001	0.956	205	<0.001
T_disgust	0.073	205	0.010	0.986	205	0.044

* This is a lower bound of the true significance. ^a^ Lilliefors Significance Correction.

**Table 3:** Total, direct, and indirect effect of Demanding Parent on OCD tendencies.

	Effect	se	t	*p*	LLCI	ULCI
Total effect of X on Y	1.0674	0.1739	6.1375	*p* < 0.001	0.7245	1.4104
Direct effect of X on Y	0.1453	0.1452	1.0006	0.3182	−0.1411	0.4317
Indirect effect(s) of X on Y:		Effect	BootSE	BootLLCI	BootULCI	
	TOTAL	0.9221	0.1461	0.6379	1.2125	
	FOG	0.1825	0.0914	0.0061	0.3712	
	DPSS_tot	0.3367	0.0847	0.1830	0.5178	
	NJRE_sev	0.4029	0.0924	0.2315	0.5958	

**Table 4:** Testing the moderation of the mediated effect of Demanding Parent mode on OCD-like tendencies.

Model Summary							
	R	R-sq	MSE	F	df1	df2	*p*
	0.7821	0.6117	177.7030	38.6008	8.0000	196.0000	*p* < 0.001
**Model**							
		coeff	se	t	*p*	LLCI	ULCI
constant		25.0308	5.3235	4.7019	0.0000	14.5321	35.5295
Demanding Parent		0.1839	0.1450	1.2688	0.2060	−0.1020	0.4699
FOG		0.2037	0.0833	2.4449	0.0154	0.0394	0.3681
DPSS		0.7482	0.1027	7.2850	0.0000	0.5456	0.9507
NJRE		0.7625	0.1188	6.4195	0.0000	0.5282	0.9967
Punitive Parent		−0.1070	0.2307	−0.4638	0.6433	−0.5621	0.3480
Int_1		0.0405	0.0162	2.5037	0.0131	0.0086	0.0724
Int_2		−0.0156	0.0209	−0.7462	0.4564	−0.0569	0.0257
Int_3		0.0240	0.0203	1.1818	0.2387	−0.0161	0.0642

**Table 5:** Conditioned effects of fear of guilt on OCD-like tendencies at punitive parenting mode values.

Focal predict: FOG (M1)						
Mod var: Punitive parent (W)						
Conditional effects of the focal predictor at values of the moderator(s):						
**Punitive Parent**	**Effect**	**se**	**t**	***p***	**LLCI**	**ULCI**
−4.9031	0.0051	0.1051	0.0485	0.9614	−0.2023	0.2124
0.0000	0.2037	0.0833	2.4449	0.0154	0.0394	0.3681
4.9031	0.4024	0.1242	3.2401	0.0014	0.1575	0.6473

**Table 6:** Effect of Demanding Parent mode mediated by fear of guilt on OCD-like tendencies at different moderator values.

Conditional indirect effects of X on Y:					
INDIRECT EFFECT:					
Demanding Parent -> FOG -> Tendencies					
	Punitive Parent	Effect	BootSE	BootLLCI	BootULCI
	−4.9031	0.0048	0.1063	−0.2077	0.2156
	0.0000	0.1923	0.0861	0.0196	0.3562
	4.9031	0.3797	0.1283	0.1162	0.6169
Index of moderated mediation:					
		Index	BootSE	BootLLCI	BootULCI
Punitive parent		0.0382	0.0164	0.0048	0.0693
Pairwise contrasts between conditional indirect effects (Effect1 minus Effect2)					
Effect1	Effect2	Contrast	BootSE	BootLLCI	BootULCI
0.1923	0.0048	0.1874	0.0804	0.0235	0.3398
0.3797	0.0048	0.3749	0.1607	0.0471	0.6795
0.3797	0.1923	0.1874	0.0804	0.0235	0.3398

**Table 7:** Effect of demanding parenting mode, mediated by disgust tendency and sensitivity, on OCD-like tendencies at different moderator values.

Demanding Parent -> DPSS ->Tendencies				
Punitive Parent	Effect	BootSE	BootLLC1	BootULC1
−4.9031	0.3624	0.1138	0.1594	0.6026
0.000	0.3287	0.0833	0.1786	0.505
4.9031	0.295	0.0868	0.1501	0.4898
Index of moderated mediation:				
Punitive Parent	Index	BootSE	BootLLC1	BootULC1
	−0.0069	0.0117	−0.0293	0.0176

**Table 8:** Effect of Demanding Parent mode mediated by NJRE severity on OCD-like tendencies at different moderator values.

Indirect Effect:				
Demanding Parent -> NJRE -> >Tendencies				
Punitive Parent	Effect	BootSE	BootLLC1	BootULC1
−4.9031	0.3361	0.1171	0.1355	0.5872
0.000	0.3976	0.0912	0.2333	0.5909
4.9031	0.459	0.1031	0.266	0.6666
Index of moderated mediation:				
Punitive Parent	Index	BootSE	BootLLCI	BootULC1
	0.0125	0.0127	−0.0137	0.0366

## Data Availability

Data will be made available on request.
